# Synthol systemic complications: Hypercalcemia and pulmonary granulomatosis. A case report

**DOI:** 10.1016/j.amsu.2021.102771

**Published:** 2021-09-03

**Authors:** Mersad Alimoradi, Ahmad Chahal, Elie El-Rassi, Karim Daher, Gazy Sakr

**Affiliations:** a Lebanese University, Faculty of Medical Sciences, Department of General Surgery, Lebanon; b Lebanese University, Faculty of Medical Sciences, Department of Radiology, Lebanon; c Lebanese American University, School of Medicine, Department of Urology, Lebanon; d Mount Lebanon Hospital, Department of Urology, Lebanon

**Keywords:** Foreign body granulomatosis, Lung granuloma, Hypercalcemia, Fillers complication, Nephrocalcinosis, Synthol, Nephrolithiasis, Body contouring, ESWL, extracorporeal shock wave lithotripsy, KUB, Kidney ureter bladder, PTH, parathyroid hormone, CT, computed tomography, PMMA, polymethylmethacrylate, PET, positron emission tomography

## Abstract

**Introduction:**

Synthol injection for body contouring has been used by bodybuilders for some time. We report two extremely rare systemic complications; pulmonary granulomatosis and hypercalcemia, in a woman who has received Synthol injections for buttocks augmentation.

**Presentation of case:**

The case discussed in this report is of a 36-year-old lady who presented for severe hypercalcemia and nephrocalcinosis. Subsequent workup revealed granulomas in the buttocks and in the lungs. Upon questioning, it was discovered that she had received Synthol injection for buttocks augmentation a few months earlier. Labs were consistent with calcitriol mediated hypercalcemia, a phenomenon observed in granulomatous diseases. A diagnosis of foreign body granulomatosis with pulmonary migration and secondary hypercalcemia was made. The patient was started on prednisone and showed an initial positive response.

**Discussion:**

Reported complications of Synthol include pain, muscle deformity, and ulceration at the injection site. Hypercalcemia secondary to foreign body granulomatosis after Synthol injection has been reported only once previously, and here we report a second case. The hypercalcemia is thought to be calcitriol mediated, where overexpression of CYP27B1 in the macrophages forming the granulomas leads to pathological extrarenal calcitriol production. Pulmonary granulomatosis, theorized to be secondary to hematologic migration of the injected material, has never been reported previously with Synthol use.

**Conclusion:**

Synthol injection for body contouring may be a cause of extensive local and pulmonary foreign body granulomatosis leading to calcitriol mediated hypercalcemia. History of cosmetic injections should not be disregarded during history taking.

## Highlights

• Synthol injections for site enhancement can lead to systemic complications
• Pulmonary granulomatosis can be secondary to hematogenic spread of Synthol
• Hypercalcemia can be secondary to severe foreign body granulomatosis
• Hypercalcemia is due to overexpression of CYP27B1 in macrophages
• Site enhancement injections should not be dismissed during history taking

## 1 Introduction

Synthol is a site enhancement oil used cosmetic contouring and volume augmentation, even though it's not approved for such use. Synthol is usually categorized along with other site enhancement oils, like paraffin, sesame, and walnut oil [1].

Reported complications after Synthol injection include pain, muscle deformity, and local ulceration [2]. Systemic complications with the use of Synthol are rarely reported. Foreign body granulomatosis with calcitriol mediated hypercalcemia has been only reported a single time previously [2], and here we report a second case. What makes this case unique is the presence of pulmonary granulomatosis, discovered incidentally during workup, theorized to be secondary to hematologic spread of the injected material.

This case was reported in accordance with the SCARE 2020 guidelines [3].

## 2 Presentation of case

A 36-year-old woman with 1 year's history of bilateral recurrent calcium oxalate stones presented to our Urology clinic for evaluation of her illness. Over the past year, she had undergone multiple sessions of extracorporeal shock wave lithotripsy (ESWL), and was admitted several times for obstructive hydronephrosis with acute kidney injury and had changed her chronic double J catheters on multiple occasions.

**Fig. 1:**
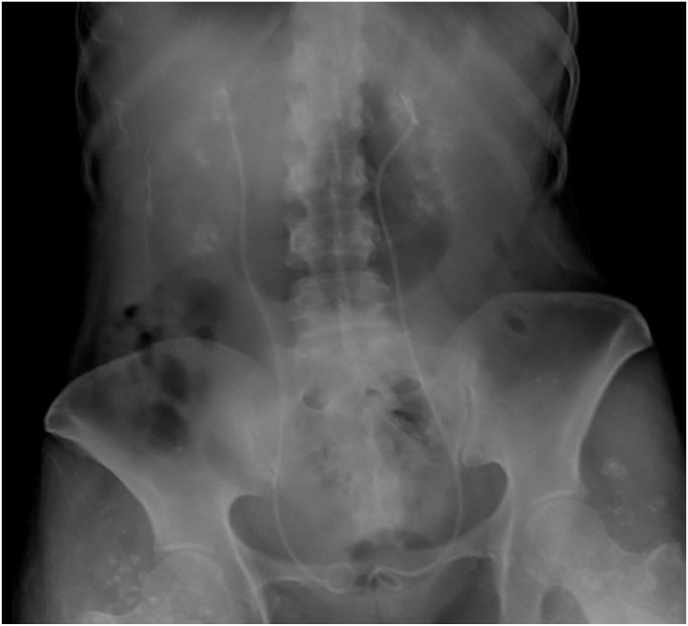
KUB X-ray showing bilateral nephrocalcinosis and the two double-J catheters in place.

Upon presentation to our clinic, she was not acutely ill. A KUB X-ray revealed bilateral nephrocalcinosis (Fig. 1). Outpatient workup revealed a serum calcium level of 12,9 mg/dL. Further hypercalcemia workup revealed a suppressed parathyroid hormone (PTH) level at 4.8 pg/ml, while 1,25-Dihydroxycholecalciferol (calcitriol) was elevated to 85 pg/ml, and 25-hydroxycholecalciferol (calcifediol) was subnormal at 18.4 ng/ml.

**Fig. 2:**
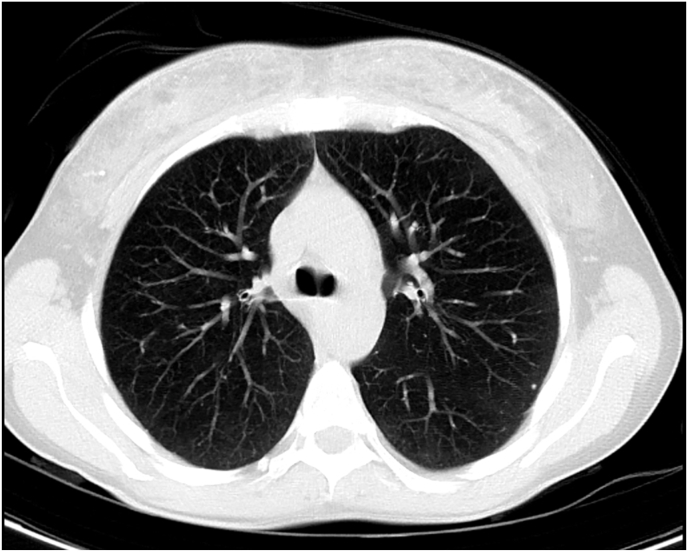
Reconstructed axial 5mm-thick maximum intensity projection (MIP) image of chest CT showing a subpleural calcified micronodule in the apico-posterior segment of the left upper lobe, one of several subpleural micronodules scattered across both lung fields.

**Fig. 3:**
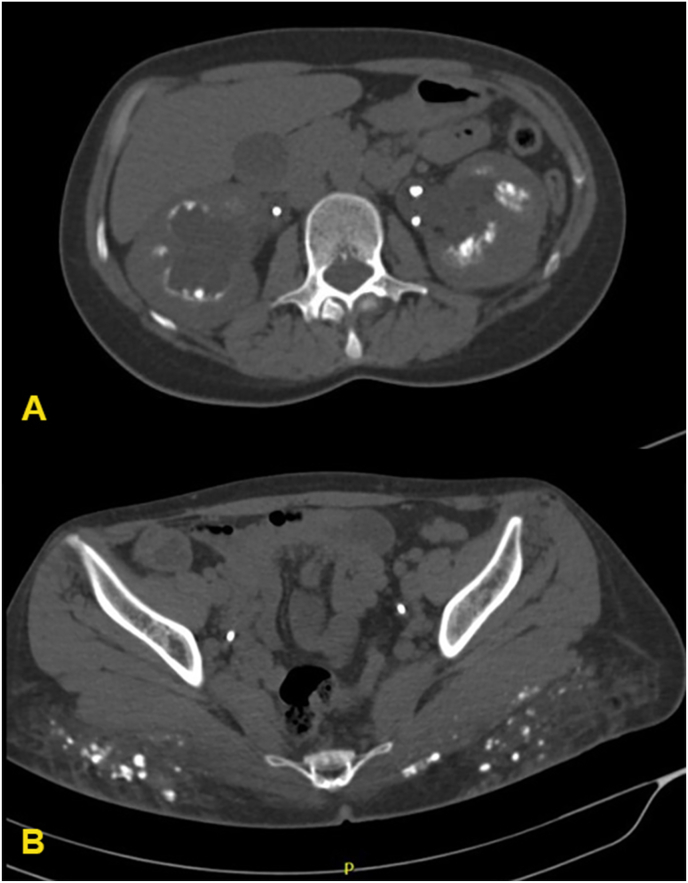
CT scan of the abdomen showing (A) bilateral nephrocalcinosis and (B) innumerable small calcifications with surrounding fat stranding in the gluteal muscles and the overlying fat plane.

Chest, abdomen, and pelvis computed tomography (CT) were ordered in search of an extrarenal source of calcitriol. Multiple small nodules (average size 4 mm) were noted dispersed in both lungs along the fissures, perivascular bundles, and subpleural region (Fig. 2) denoting a possible granulomatous disease. The kidneys showed clusters of medullary pyramidal calcifications consistent with nephrocalcinosis with multiple small pelvic, ureteral, and bladder stones (Fig. 3A). Interestingly, the radiologist noted innumerable small calcifications with surrounding fat stranding in the gluteal muscles and the overlying fat plane (Fig. 3B) and an enlarged right inguinal lymph node.

During follow-up, further questioning was done, and the patient revealed that she had undergone injections of Synthol fillers in the gluteal region for cosmetic augmentation a few months before her problems started. A diagnosis of local (gluteal) and pulmonary foreign body granulomatosis with secondary hypercalcemia and nephrocalcinosis was suspected. To confirm the diagnosis, an excisional biopsy of the enlarged inguinal lymph node was performed, and the pathology demonstrated granulomatous lymphadenitis most consistent with a foreign body reaction.

The patient was referred to an endocrinologist and was started on oral prednisone 20 mg daily. Her calcium levels remained elevated after a month of follow up, so the dose was increased to 40 mg daily. A month later, and at the time of writing of this report, the calcium level decreased and was close to the upper limit of normal at 10.6 mg/dL.

## 3 Discussion

Dating back to 1894, site enhancement oils were first used as fillers to remove facial wrinkles and augment certain body parts, such as the breasts. Synthol, a famous site enhancement oil, has been used by bodybuilders since 1996 to augment muscle size [4]. It is composed of 85% oil (medium-chain triglycerides), 7.5% lidocaine, and 7.5% alcohol.

Synthol is not an approved injectable filler, however, it is still used by some for cosmetic augmentation and contouring. Local deformity, pain, ulceration, and foreign body granulomatosis are all reported complications of Synthol injection [1,4,5].

Systemic complications after Synthol injection are, however, rare. Hypercalcemia has only been previously reported once with Synthol use [2], but pulmonary complications have never been reported before. This is part of what makes the above-presented case unique.

Although never reported with Synthol, serious, and sometimes fatal, pulmonary complications have been reported as a complication of other cosmetic injectables (like silicone) [6].

The pulmonary complications include acute pneumonitis, pulmonary embolism, acute pulmonary hemorrhage, acute granulomatous pneumonitis, and diffuse alveolar damage. Chronic pulmonary granulomatosis associated with soft-tissue filler injection has only been reported once previously in 2012 [6]. The entity may be under-reported in the literature, since some patients might have asymptomatic pulmonary granulomatosis, like our case, and never get diagnosed. We do believe, however, that our patient falls within the same spectrum of the other reported cases, and would eventually develop lung manifestations.

Pulmonary complications related to soft-tissue fillers are thought to be a result of hematologic migration of the injected material due to high tissue pressure at the injection site, accidental intravenous injection of the material, or massage at the injection site [6].

Pulmonary granulomatosis occurs when insoluble particles migrate to the lung and lodge in the pulmonary arteries causing initial arteritis [7]. The particles are phagocytosed by macrophages and giant cells as they cross to the interstitium, causing small nodularities, predominantly in a perihilar and upper lobe distribution.

In regards to hypercalcemia, Chiri et al. reported a case similar to ours in 2017 [2], where a young bodybuilder developed foreign body granulomatosis after Synthol injection, leading to calcitriol mediated hypercalcemia and subsequent pancreatitis and nephrocalcinosis.

Foreign body granulomatosis can lead to hypercalcemia due to an overexpression of CYP27B1 in the macrophages forming the granuloma, leading to pathological extrarenal calcitriol production [8].

Hypercalcemia can present with non-specific symptoms of fatigue, lethargy, abdominal pain, constipation, neurologic dysfunction, weight loss, and renal complications. In their systematic review published in 2018 [8], Tachamo and colleagues reported that renal failure was the most common complication (82%) in patients who had hypercalcemia secondary to a granulomatous reaction to injected soft-tissue fillers. The review had identified 23 cases of hypercalcemia, occurring months to years after the injection, with a mean of 8 years. Silicone was reported as the most commonly used agent. In two of the 23 reported cases, surgical excision of the granulomas was attempted, however, unsuccessfully due to extensive granulomatosis [8].

Diagnosis can usually be achieved by combining laboratory findings of calcitriol mediated hypercalcemia, imaging with CT, PET, or gallium scanning, and biopsy of the involved tissue. Biopsies typically show multinucleated giant cells forming a granulomatous reaction, with the expression of CYP27B1 [7,8]. Usually, further interventions, such as bronchoscopy and lung biopsies, are not needed unless the diagnosis is still unclear.

Current treatment options include corticosteroids and bisphosphonates, with sparse reports of the use of denosumab, calcitonin, and ketoconazole. Despite medical treatment, almost half of the patients develop recurrent hypercalcemia [8].

## 4 Conclusion

Although rarely reported, Synthol is capable of producing systemic complications like the ones reported in this case. Granulomatosis at the injection site, pulmonary granulomatosis, hypercalcemia, and renal failure can all occur. The etiology is worth keeping in mind when assessing a patient with relevant history. Site enhancement injections should not be dismissed during history taking as they can be critical to the diagnosis.

## Sources of funding

This research did not receive any specific grant from funding agencies in the public, commercial, or not-for-profit sectors.

## Ethical approval

The study type is exempt from ethical approval.

## Consent

Written informed consent was obtained from the patient for publication of this case report and accompanying images. A copy of the written consent is available for review by the Editor-in-Chief of this journal on request.

## Author contribution

Mersad Alimoradi was responsible for design and writing of the first draft of the article. Elie El-Rassi and Karim Daher were responsible for data collection and patient follow up. Ahmad Chahal was responsible for imaging interpretation and analysis. Ghazi Sakr supervised the work and provided the required corrections to obtain the final draft.

## Research registration

N/A.

## Guarantor

Dr. Ghazi Sakr, M.D.

## Provenance and peer review

Not commissioned, externally peer-reviewed.

## Declaration of competing interest

The authors report no conflict of interest with any parties in regards to this research article.
